# Gun-Shot Wound—Delirium Tremens, Administration of Chloroform in, Etc.

**Published:** 1867-11

**Authors:** L. H. Orme

**Affiliations:** Atlanta, Ga.


### ARTICLE II.

*Gun Shot Wound—Delirium Tremens, Administration of Chloroform in, &c.* By L. H. Orme, M. D., Atlanta, Ga.

About six o'clock on the afternoon of the first of January, 1866, I was requested to see Mr H., of this city, who had been the innocent, but unfortunate victim of what apparently seemed to be a very trifling, but which subsequently proved to be a very dangerous gun shot wound.

Upon a thorough examination I found that the ball (fired, judging from the character of the aperture, from a Colt's Repeater) struck immediately upon the outer aspect of the Great Trochanter—indenting the bone—and in all probability fell back through the wound it had made, as a few days later a ball was found among the clothing of the patient, supposed to be the same by which the injury was inflicted, and as the most careful probing would discover no trace of the ball in that direction I became settled in the conviction that it had made its exit in this way.

My patient experienced no considerable inconvenience until the next morning at nine o'clock, when he complained of great pain in the region of the wound and in the whole extent of the leg.

In answer to his urgent request some stimulant was administered very sparingly and with a view to alleviating his great suffering, which was now becoming very intense; a grain of Morphine was also administered. This succeeded in relieving him temporarily, but it was apparent that the power of his whole nervous system was rapidly becoming entirely dethroned and the further exhibition of stimulants or powerful nervous agents would not only fail in relieving the difficulty but would prove positively injurious by augmenting and continuing this deranged condition, which on the third or fourth day began to develope unmistakable symptoms of Delirium Tremens.

Previous to the reception of the injury this patient had been on no debauch or "spree," as it is usually termed—had been accustomed, however, to a moderate indulgence in alcoholic stimuli—and was in good health.

Supervening upon this injury we have a case of Delirium Tremens, as well marked as any ever known to the profession, which certainly could have been the result of nothing but "shock" from mechanical injury with subsequent failure properly to reäct.

Dr. R. C. Word, of this place, was called in consultation, and the treatment usually adopted in such cases resorted to without any beneficial results.

There was no attempt on the part of the patient to quit his bed, nor did he make any violent demonstrations other than boisterous language at times.

He was exceedingly annoyed by the continual presence of snakes crawling on the wall and about his bed, and frequently called upon different members of his family to remove lizzards from the "mantle piece" as well as other reptiles and little animals in his judgment not sufficiently domesticated to mingle in his household.

Upon the suggestion of Dr. Word, the Elixir of Opium and small quantities of brandy were administered in order to procure sleep; but our patient successfully resisted all such medication, and in spite of our efforts to relieve him this distressing condition of things had now extended to the ninth day, and it was very evident that the patient's physical system was fast succumbing under the influence of this terrible excitement, and that unless rest could speedily be obtained the last vestige of hope would disappear.

Upon consultatiou with Drs. W. F. Westmoreland and J. F. Alexander, of this city, I decided, as a *dernier* resort, to place him profoundly under the influence of Chloroform—and I must confess it was not without some misgiving that I carried this into execution, as the nutritive process had been suspended for so long a time, and vitality was at so low an ebb, it was extremely problematical how far the contemplated soporific influence would extend.

Chloroform was administered about 4 o'clock in the afternoon and carried to complete anesthesia, which lasted until 1 o'clock the following morning, when he awoke and spoke rationally, to his brother who was sitting near, (memorable words with his family) for the first time in nine days.

I am firmly persuaded that had it not been for the exhibition of Chloroform in this instance the patient must have inevitably perished.

He had no return of delirium from that time and continued to improve under a dietetic treatment until he was finally restored to perfect health.

Dr. Bennet, in his excellent work on the practice of medicine, recommends that in Delirium Tremens all medication should be dispensed with and an entirely nutrient treatment adopted, and gives a tabular statement of twenty cases admitted to the hospital of which he had supervision, during the months of May, June and July of 1864, all of whon recovered under a simply dietetic treatment with the exception of two, to whom at the outset, emetics were administered.

The average duration of these cases, or at least of their stay in the hospital, being about six days.

I am disposed to agree with the author that supplying the accustomed stimulus, theoretically considered, is tantamount to adding coals to fire, and practically, experience teaches that patients more rapidly recover under a nutrient treatment, *as a general rule*, and provided their systems will appropriate the nutrition taken into the stomach; but then I contend there are cases, as the one related above, in which to depend upon such a course were simple madness; for whilst you were waiting for the assimilation of your nutrients the exhaustive process of the disease would terminate your treatment in the death of your patient.

